# Microvascular Injury in Ketamine-Induced Bladder Dysfunction

**DOI:** 10.1371/journal.pone.0160578

**Published:** 2016-08-16

**Authors:** Chih-Chieh Lin, Alex Tong-Long Lin, An-Hang Yang, Kuang-Kuo Chen

**Affiliations:** 1 Institute of Clinical Medicine, National Yang-Ming University, Taipei, Taiwan; 2 Department of Urology, School of Medicine, National Yang-Ming University, Taipei, Taiwan; 3 Department of Urology, Taipei Veterans General Hospital, Taipei, Taiwan; 4 Department of Pathology and Laboratory Medicine, Taipei Veterans General Hospital, Taipei, Taiwan; 5 Department of Pathology, School of Medicine, National Yang-Ming University, Taipei, Taiwan; Cedars-Sinai Medical Center, UNITED STATES

## Abstract

The pathogenesis of ketamine-induced cystitis (KC) remains unclear. In this study, bladder microvascular injury was investigated as a possible contributing mechanism. A total of 36 KC patients with exposure to ketamine for more than 6 months, and 9 control subjects, were prospectively recruited. All participants completed questionnaires, including the O’Leary–Sant interstitial cystitis symptom index (ICSI) and the interstitial cystitis problem index (ICPI). All KC patients received a urodynamic study and radiological exams. Bladder tissues were obtained from cystoscopic biopsies in the control group and after hydrodistention in the KC group. Double-immunofluorescence staining of *N*-methyl-d-aspartate receptor subunit 1 (NMDAR1) and the endothelial marker, cluster of differentiation 31 (CD31), was performed to reveal the existence of NMDAR1 on the endothelium. Electron microscopy (EM) was applied to assess the microvascular change in the urinary bladder and to measure the thickening of the basement membrane (BM). A proximity ligation assay (PLA) was used to quantify the co-localization of the endothelial CD31 receptor and the mesenchymal marker [fibroblast-specific protein 1 (FSP-1)]. The Mann–Whitney U test and Spearman’s correlation coefficient were used for statistical analysis. The mean ICSI [14.38 (± 4.16)] and ICPI [12.67 (± 3.54)] scores of the KC group were significantly higher than those (0 and 0, respectively) of the control group (both *p* < 0.001). The KC patients had decreasing cystometric bladder capacity (CBC) with a mean volume of 65.38 (± 48.67) mL. NMDAR1 was expressed on endothelial cells in both groups under immunofluorescence staining. Moreover, KC patients had significant BM duplication of microvessels in the mucosa of the urinary bladder under EM. The co-expression of the endothelial marker CD31 and mesenchymal marker FSP1 was significantly stained and calculated under PLA. In conclusion, microvascular injury and mesenchymal phenotypic alteration of endothelial cells can potentially contribute to KC-induced bladder dysfunction.

## Introduction

Ketamine, considered pharmacologically as a non-competitive *N*-methyl-d-aspartate (NMDA) receptor antagonist, has been used clinically since 1970 in dissociative anesthesia for elective surgeries of infants and children [1,2]. Gonzalez-Cadavid et al. noted the presence of NMDAR in urogenital organs, including penile, prostate, and human bladder [3]. In recent years, ketamine has been used illicitly for recreational purposes, leading to catastrophic effects on the health of young adults, including not only cognitive disorder but also devastating urological sequelae (e.g., diminished bladder capacity). Because of ease of accessibility and lower expense relative to other addictive drugs, the problems of ketamine abuse are getting worse in many Asian countries [4]. Since Shahani et al. first reported that patients with ketamine-induced cystitis (KC) can display severe dysuria, hematuria, and urinary frequency, many researchers have proposed possible pathogenic mechanisms for these signs and symptoms [4–9]. Most KC patients start to suffer from severe urinary tract symptoms after long-term nasal administration, rather than smoking. The most common histological feature in KC patients is chronic inflammation, associated with mucosa ulceration, and infiltration of neutrophils and lymphocytes [4,5,7,9]. Fibrotic change of the urinary bladder after chronic inflammation may be a cause of diminished bladder capacity, leading to severe urinary frequency with extremely short intervals [10]. In the brain, the NMDA receptor subunit 1 (NMDAR1) is expressed in brain endothelial cells; it regulates tissue-type plasminogen activator-induced signal transduction and controls the passage of monocytes through the brain endothelial cell barrier [11]. In the urinary bladder, the association between chronic inflammation and NMDAR1 is less well established. Chu et al. proposed that ketamine might induce microvascular changes in the bladder, causing endothelial cell injury of microvessels and subsequent compromised intrinsic microcirculation [5]. “Gross hematuria” can also be an important symptom in patients with KC, with “increasing vascular distribution” as a cystoscopic finding [5,7–9,12]. Similar to brain and other organ systems, endothelial cells of the urinary bladder might be possible targets of microvascular injury (MVI) and result in fibrosis during ketamine abuse [11,13].

The aim of this study was to confirm the presence of NMDAR1 in the urinary bladder and, using electron microscopy, determine the ultrastructural changes of the endothelial cells during microvascular injury after ketamine abuse.

## Materials and Methods

The study was approved by the Institutional Review Board and Ethics Committee of Taipei Veterans General Hospital (IRB number: 201008025GB). After fully informing them of the study rationale and procedures, each written informed consent was obtained prior to performing the invasive procedures. All KC patients were prospectively recruited under the criteria of having lower urinary tract symptom (LUTS) and having regularly consumed ketamine for at least 6 months, but without previously documented urological disease, including urinary tract infection or malignancy. Those patients with major systemic disease, urinary tract infection, neurological disease, diabetes, and/or a post-void residual volume of greater than 150 mL were also excluded from the KC group. All KC patients were investigated with questionnaires, including the O’Leary–Sant interstitial cystitis symptom index (ICSI) and the interstitial cystitis problem index (ICPI) [14]. In addition to the 36 KC patients enrolled in this study, 12 volunteers having other diseases, including upper urinary tract stones or cancer (but without any urgency and frequency symptoms), were also screened with the ICSI and ICPI. After excluding those with ICSI and ICPI scores of greater than 0, only nine of the volunteers were recruited to form the control group. The daily consumption of a ketamine dose, the cumulative duration of exposure to ketamine, and the interval between ketamine usage and the development of LUTS were also recorded. All patients underwent at least one imaging study (intravenous pyelography, ultrasonography, or computed tomography) to evaluate their upper urinary tracts.

After being recruited, all KC patients underwent cystoscopic hydrodistention at an intravesical pressure of 80 cmH_2_O under general anesthesia for 5 min. The maximum bladder capacity (MBC) and the degree of glomerulation after hydrodistension were recorded. The severity of glomerulation was graded from 0 (no glomerulation) to 4 (most severe glomerulation or waterfall bleeding) [15]. After hydrodistension, at least three pieces of cold-cup bladder biopsies were taken from the bladder mucosa and submucosa of the suspected lesions. These samples were subsequently prepared for electron microscopy, immunofluoresence staining, and haematoxylin and eosin staining in the Pathology Department, confirming that they were benign lesions. The cystoscopic bladder biopsies from the control group were obtained, without hydrodistention, before performing other endoscopic procedures, under general or spinal anesthesia.

**Fig 1:**
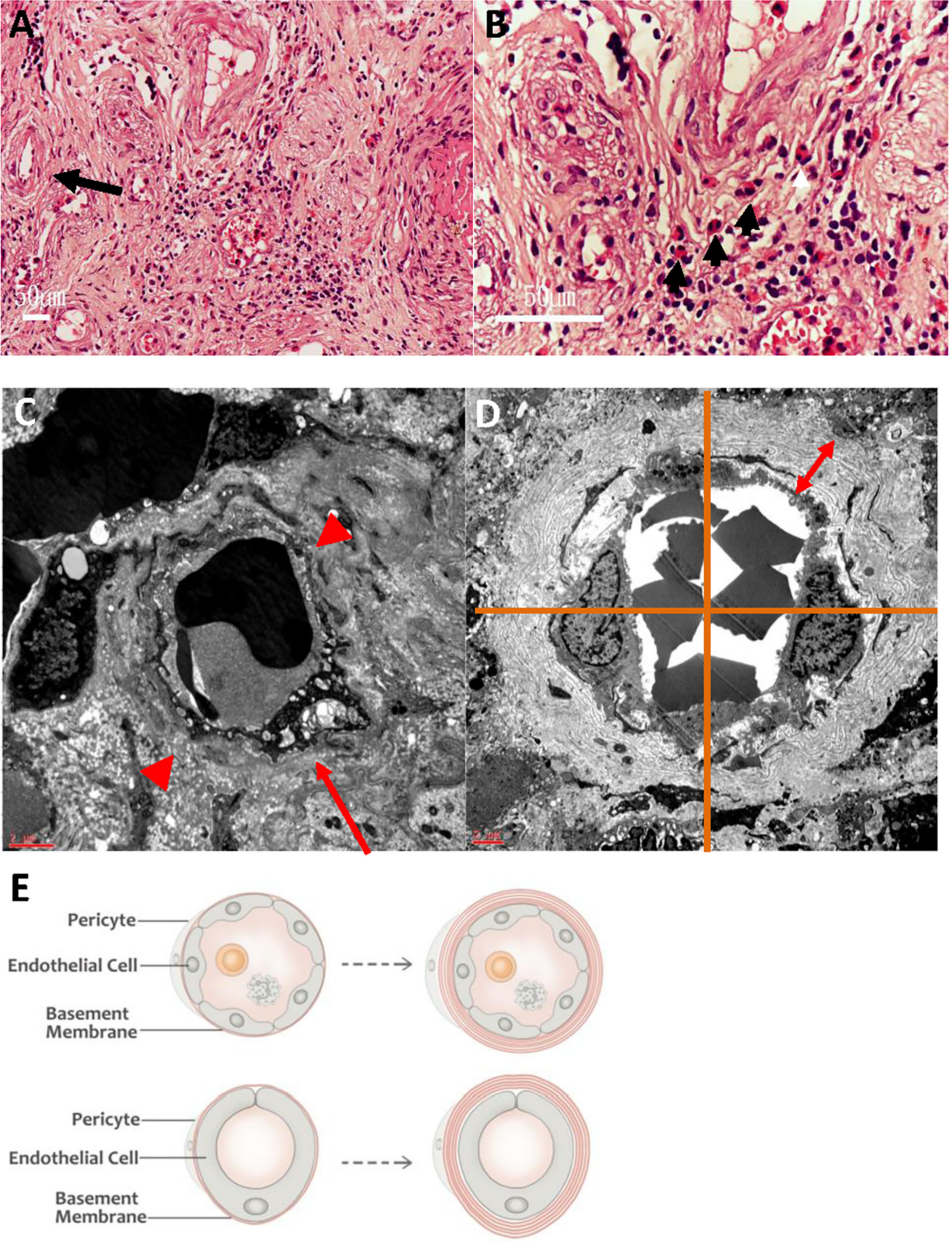
Microvascular remodeling in the urinary bladder. (A, B) Histology of bladder biopsy of patient with KC reveals many eosinophils (black arrowheads) and vascular wall thickening (long black arrow) in suburothelial tissue (hematoxylin and eosin stain): (A) reduced from x 100; (B) reduced from x 400. (C) Small vessel from a normal control subject revealing an endothelial cell (long red arrow) with a single layer of BM (red arrowheads). (D) Small vessel from a KC patient revealing onion-skin duplication of the BM (red double-ended arrow); the increasing thickness of the BM was measured after division into four quarters, using two straight lines intersecting at the center of the vascular lumen. (E) Schematic representation of the duplication of the BM in greater (upper panel) and small (lower panel) vessels. (A, B) White bars: 50 μm; (C, D) red bars: 2 μm.

Bladder biopsy tissues for transmission electron microscopy (TEM) were fixed in 2.5% glutaraldehyde and then processed according to routine protocols. After fully surveying the whole slide under thick sectioning, three locations featuring the most affected vessels were chosen for thin sectioning. The thin sections were viewed and recorded digitally using a transmission electron microscope (JEM-1230, JEOL, Japan) equipped with a multiscan CCD camera (791 MSC, Gatan, USA). The morphological difference in microcirculation in the urinary bladder was further evaluated. According to the findings of Miodonski et al., vessels having dimensions of less than 50 μm were categorized as “small vessels and capillaries,” while vessels larger than 50 μm were grouped as “greater vessels” [16]. To provide data from only a few tissue sections that could be considered representative of the tissue as a whole, steps were taken throughout the entire experimental process to ensure random sampling. These steps included selecting thin sections and assigning the KC and control groups a random accession number for each thin section to ensure that the ultrastructural analysis was performed in as blinded a manner as possible; in addition, TEM images were taken of every microvessel. After obtaining images from each section under TEM, the increasing thickness of the BM was measured by dividing the vascular lumen into four quarters, using two straight lines intersected at the center (Fig 1D). More than five microvessels from each of the thin sections were recorded; all of the final data for the mean increasing thickness from the four quarters were measured blindly by C.C. Lin.

The bladder specimens were placed in pre-labeled base molds filled with frozen tissue matrix, and then cut into 5-μm-thick slices for frozen sectioning. As in the routine procedures, the section slides were fixed by immersion in cold acetone (–20°C) for 2 min and then dried at room temperature. To examine the co-expression of NMDA receptor 1 (NMDAR1) and the cluster of differentiation 31 (CD31) receptor on the vessel, double-immunofluorescence staining was performed by incubating the sections overnight at 4°C with the following primary antibodies (Novus Biologicals, Littleton, USA): anti-human CD31 antibody (mouse, 1:20; catalog code: NB600-562) and anti-human NMDAR1 (rabbit, 1:50; catalog code: NB100-74473). The co-localization of endothelial CD31 receptor and the two mesenchymal markers—fibroblast specific protein 1 (FSP-1; rabbit, 1:100; Abcam, Cambridge, UK; catalog code: ab27957) and α-smooth muscle actin (α-SMA; rabbit, 1:200; Abcam, Cambridge, UK; catalog code: ab5694)—was performed using double-immunostaining. All sections were examined under an Olympus Vanox-s AH-2 immunofluorescence microscope equipped with a Micrometrics® 590CU digital camera. ImageJ software (v. 1.37c, USA) was used to process the double-immunofluorescence images.

To precisely quantify the co-expression of endothelial and mesenchymal markers, the Duolink In Situ assay (Olink Bioscience, Uppsala, Sweden) was applied [17–19]. The frozen sections were incubated overnight at 4°C with a mixture of primary antibodies, including anti-human CD31 antibody (mouse, 1:20; catalog code: NB600-562) and FSP-1 (rabbit, 1:100; Abcam, Cambridge, UK; catalog code: ab27957), after incubation with blocking buffer solution (Dako, Protein Block Serum-Free). The sections were then mixed with two PLA probes—Duolink In Situ PLA probe anti-Mouse PLUS (1:10) and anti-Rabbit MINUS (1:10)—for 2 h. According to the subsequent protocol, hybridization, ligation, amplification, and detection were performed in sequence. The rolling circle products were visualized, using fluorescently labeled oligonucleotides, as fluorescent red dots. Duolink ImageTool software (Olink Bioscience, Uppsala, Sweden) was used for image analysis. Negative controls included the isotypes of the primary antibodies. The mean, maximum, range, and standard deviation (SD) of the PLA signal dots of endothelial cells were recorded.

Statistical analysis was performed using SPSS for Windows (v. 14.0, SPSS, Chicago, IL). The Mann–Whitney U test was applied to compare continuous variables between the two groups. Fisher’s exact test was used to compare the category variables. All clinical parameters were also correlated with the increased thickness of BM by using Spearman’s correlation coefficient. Differences were considered statistically significant when *p* was less than 0.05.

## Results

Sixteen (44.4%) men and twenty (55.6%) women constituted the KC patients group, with a mean age of 27 years (range: 20–40 years); they were significantly younger than the control (*p* < 0.001). None of the KC patients had a history of acute or chronic hepatitis or liver cirrhosis. Ten patients (27.8%) were found to have abnormal liver function tests and one (2.7%) even had jaundice. As listed in Table 1, the KC patients had consumed high dosages of ketamine daily; most of them had started to have troublesome LUTS 1.5 years after they had begun abusing. The mean cumulative duration of exposure to ketamine was 3.56 years. From the questionnaires, the KC group had severe symptom scores, with median ICSI and ICPI scores of 14.50 and 13.00, respectively (Table 2). From radiological examinations, the most common abnormality was hydronephrosis, followed by vesicoureteral reflux. During hydrodistention, the mean maximum bladder capacity (MBC) under anesthesia of the KC patient group was 218.08 mL (± 119.00). After hydrodistention, most of the KC patients exhibited grade 3 and 4 changes in the urinary bladder (Table 1). No complications (e.g., excessive bleeding; perforation) occurred after the bladder biopsies. Histological findings of the biopsies taken from all of the KC patients revealed submucosal inflammation with eosinophil infiltration (Fig 1A and 1B).

**Table 1:** Demographic description of KC patient groups.

	KC
**Number**	*N* = 36
**Gender (M:F)**	16:20
**Mean age (years)**	26.89 (± 5.05)
**Ketamine consumption**	
**Mean daily dosage (gram)**	4.75 (± 1.14)
**Mean cumulative duration of exposure to ketamine (years)**	3.56 (± 1.83)
**Mean interval from initial consumption to LUTS (years)**	1.60 (± 1.23)
**Bladder capacity in hydrodistention**	**Mean (± S.D.)**
**Mean MBC (mL)**	218.08 (± 119.00)
**Cystoscopic finding after hydrodistention**	***N* (%)**
**Mean overall glomerulation grade (± S.D.)**	3.22 (± 0.99)
**Normal appearance**	1 (2.8%)
**Hypervascularity (grade 1)**	1 (2.8%)
**Diffuse glomerulation, scattered submucosal ecchymosis (grade 2)**	5 (13.9%)
**Waterfall bleeding with diffuse submucosal ecchymosis (grade 3)**	11 (30.6%)
**Cracks or fissures into muscle layer (grade 4)**	18 (50%)

CMG, cystomyogram; IC/PBS, interstitial cystitis/painful bladder syndrome; KC, ketamine-induced cystitis; LUTS, lower urinary tract symptoms; MBC, maximal bladder capacity.

**Table 2:** Demographic and cystoscopic findings in the control and KC patient groups.

	Control (*n* = 9)	KC (*n* = 36)	*p*
**Gender (M:F)**	4:3	16:20	<0.0001
**Mean age (± SD)**	48.43 (± 12.21)	26.89 (± 5.05)	0.0168
**Hydronephrosis**	0% (0/9)	22.22% (8/36)	0.179
**Vseico-ureteral reflux**	0% (0/9)	16.67% (6/36)	0.323
**Median ICSI (Minimal, Maximal)**	0 (0,0)	14.50 (5, 20)	<0.0001
**Median ICPI (Minimal, Maximal)**	0 (0,0)	13.00 (2, 20)	<0.0001

ICSI, interstitial cystitis symptom index; ICPI, interstitial cystitis problem index.

**Fig 2:**
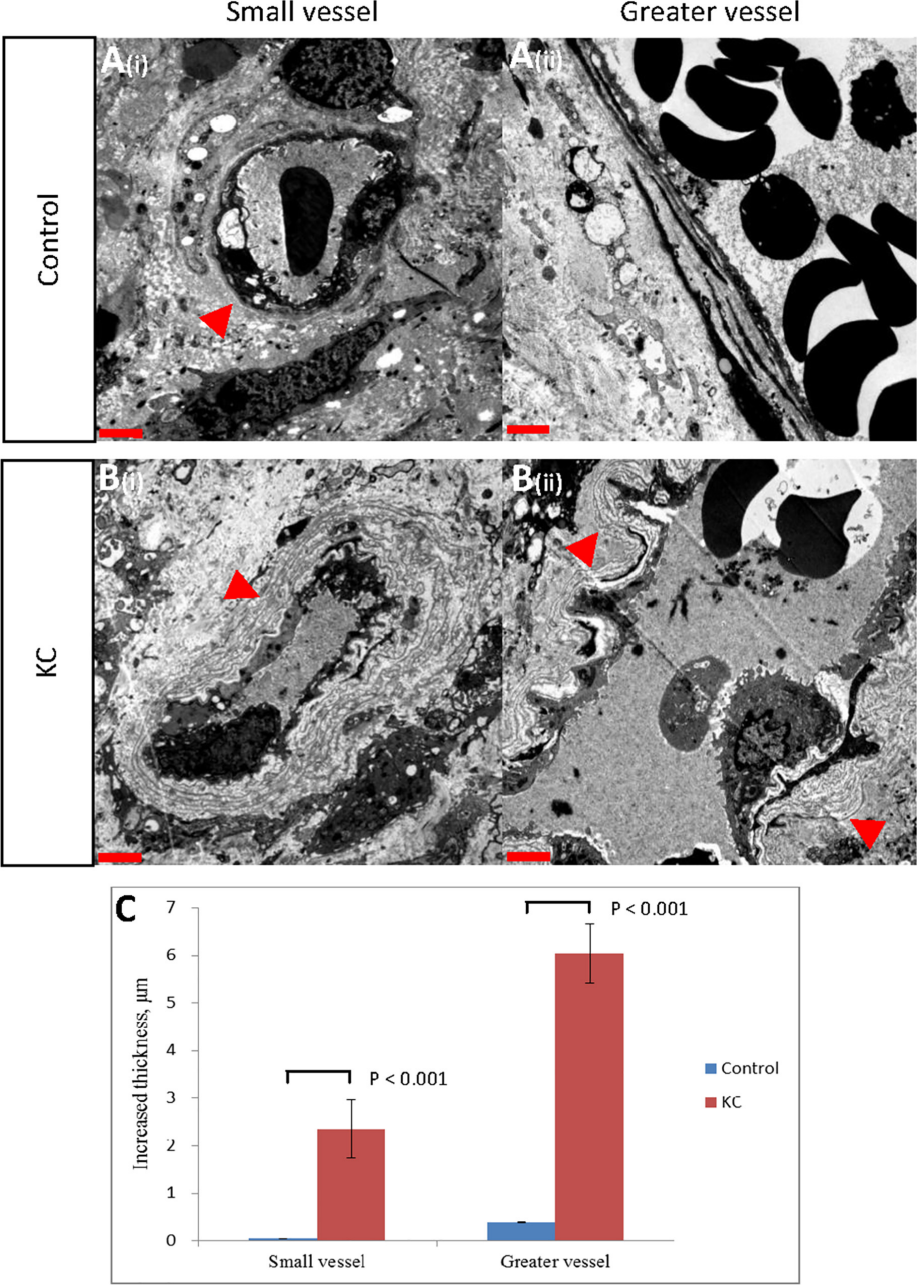
Comparison of microvascular remodeling in the urinary bladders of control and KC patients under electron microscopy. (A) Controls, BM (arrowheads): (i) small and (ii) greater vessels. (B) KC, duplication of BM (arrowheads): (i) small and (ii) greater vessels. (C) Bar graph of the increased thickness (μm) of BM in the KC (*n* = 36) and control (*n* = 7) groups: (i) small and (ii) greater vessels; obtained using the Mann–Whitney U test for statistical analysis. (A–C) Red bars: 2 μm.

**Fig 3:**
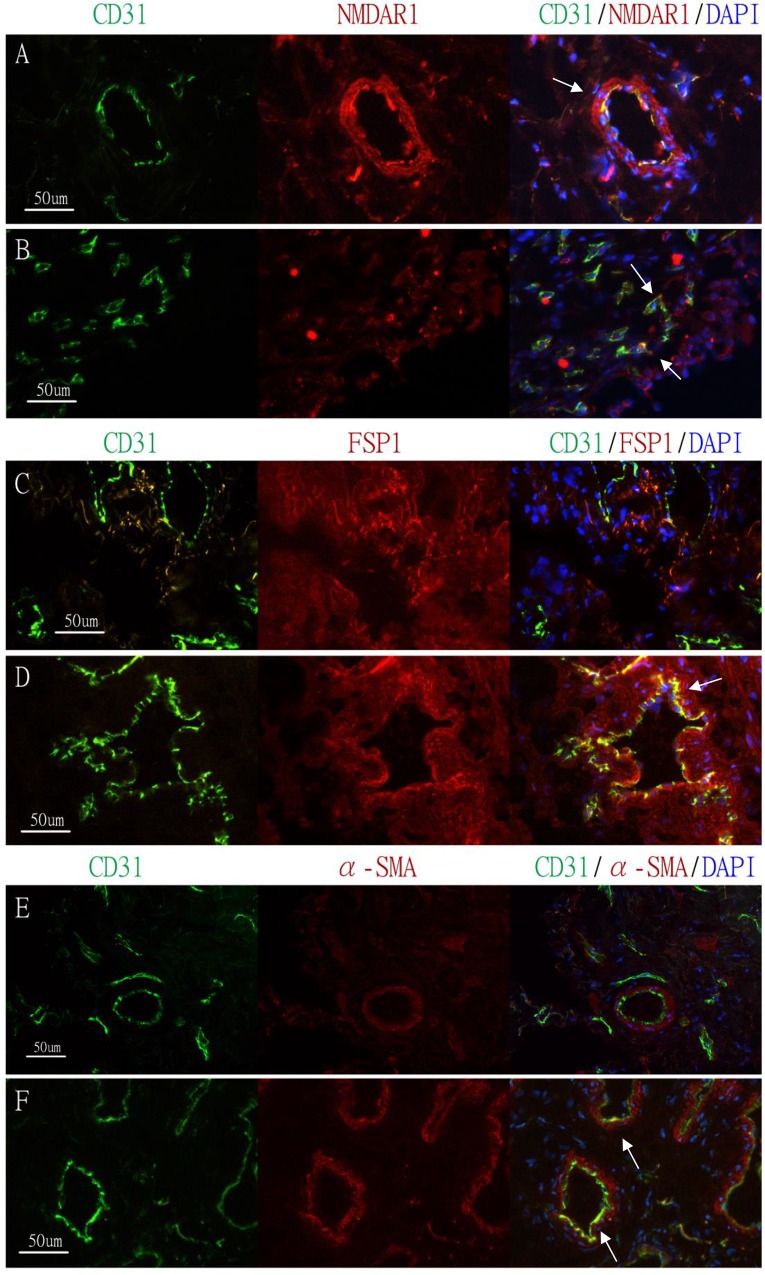
Immunohistochemial comparison of the expression of NMDAR1 on vessels and EndoMT markers in the urinary bladder. Biopsy specimens of frozen sections from the urinary bladders of (A, C, E) normal control subjects and (B, D, F) KC patients. (A, B) Double-immunofluorescence staining with antibody to endothelial marker CD31 (green) and NMDAR1 (red); DAPI was used as a nuclear stain (blue); co-expression of CD31 and NMDAR1 was evident in two groups (yellow, denoted by white arrows). (C–F) Double-immunofluorescence staining with antibody to endothelial marker CD31 (green) and two mesenchymal markers: FSP1 (red, in panels C and D) and α-SMA (red, in panels E and F); DAPI was used as a nuclear stain (blue); co-expression of CD31 and FSP1 or α-SMA was evident in three groups (yellow, denoted by white arrows) (white bars: 50 μm).

The distinct findings and morphological differences of the vessels were noted under TEM imaging (Fig 2). The BM beneath the endothelial cells was significantly thicker in the KC patient group; it had a multilayer appearance. The mean (SD) increased thickness of small and greater vessels in the KC patients were 2.34 (1.36) and 6.04 (3.70) μm, respectively—significantly greater than the values in the control group [0.04 (0.02) and 0.39 (0.38) μm, respectively] (*p* < 0.001). In addition to the findings from the TEM images, the vessels of the KC patients had tortuous and multiple angular shapes; disparately, those of the control patients had oval or round shapes (Fig 3). In Table 3, no significant correlation exists between the increased thickness of the greater or small vessels and the other parameters (i.e., the questionnaire results, the duration or daily dosage of ketamine consumption, or the bladder capacity of hydrodistention).

**Table 3:** Correlation between increased thickness of BM of vessels and clinical parameters, obtained using Spearman’s correlation coefficient for statistical analysis.

Parameter	Greater Vessel		Small Vessel	
	Correlation Coefficient	*p*	Correlation Coefficient	*p*
**Age at visit**	–0.120	0.486	0.089	0.607
**Daily ketamine dosage**	0.050	0.779	–0.116	0.515
**Interval between ketamine usage to LUTS**	–0.016	0.926	0.046	0.794
**Total duration of ketamine usage**	–0.083	0.640	0.057	0.749
**Hydrodistention capacity**	0.191	0.266	–0.052	0.764
**ICSI**	–0.019	0.928	–0.074	0.720
**ICPI**	–0.028	0.893	–0.152	0.458

ICSI, interstitial cystitis symptom index; ICPI, interstitial cystitis problem index; LUTS, lower urinary tract symptoms.

Fig 3 displays the co-localization of the NMDA receptor 1 (NMDAR1) and the CD31 receptor in the KC patients and the control group. NMDAR1 was expressed in the vascular endothelium located in the submucosa (Fig 3). Both groups featured the co-expression of CD31 and NMDAR1 in the vessels. In particular, the vessels in the FITC figure for the KC patient group were smaller in diameter than those for the control group. In Fig 3, the co-localization of the endothelial marker CD31 receptor and the mesenchymal marker FSP1 in the urinary bladders of the KC patients was stronger than that in the control group. Similarly, co-localization of CD31 and the α-SMA receptor was clearly evident in the vessels from the urinary bladders of the KC patients, but not in those from the control group.

**Fig 4:**
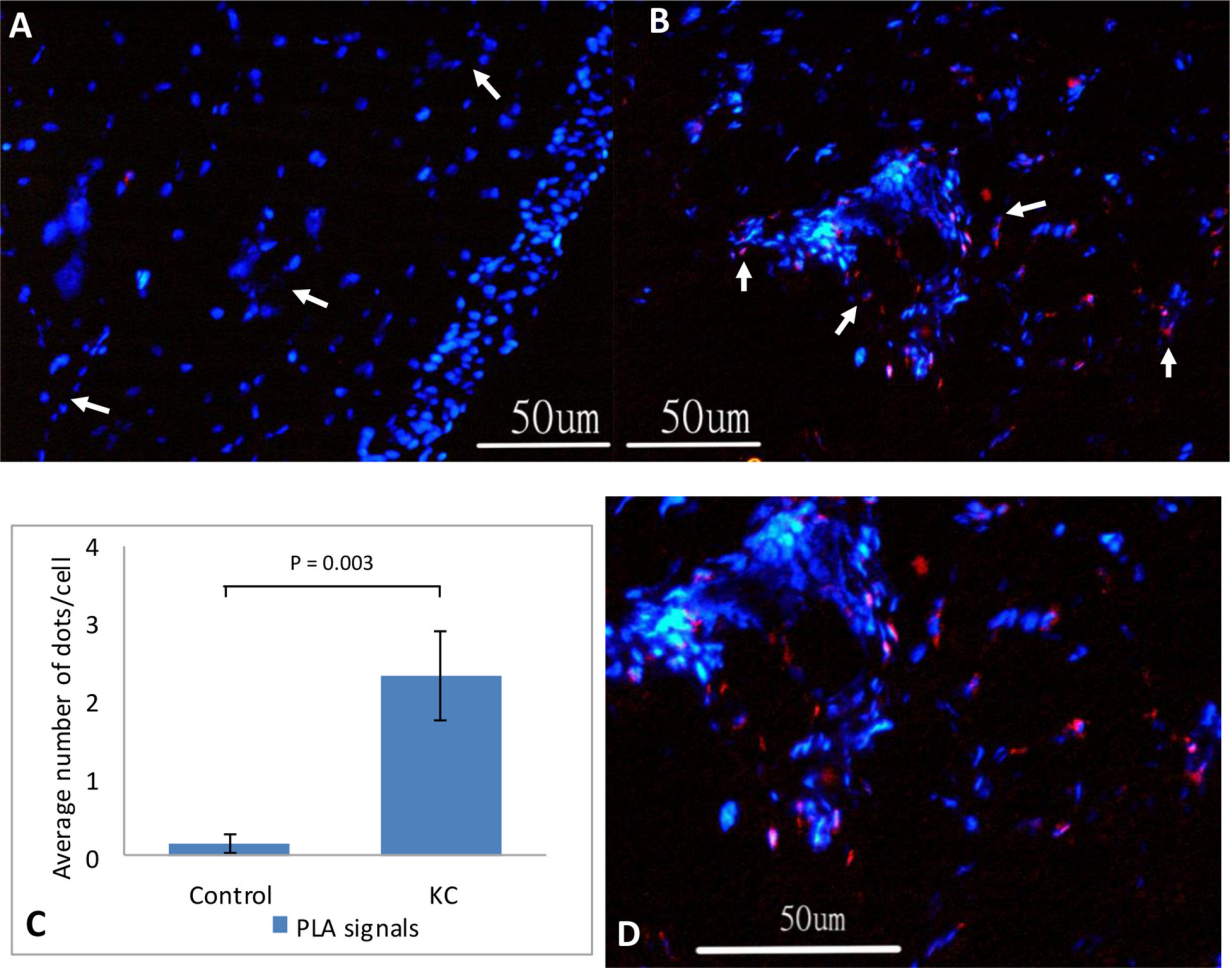
Quantitative PLA assay of co-expression of mesenchymal transition markers in urinary bladder. (A, B, D) Red PLA signal dots represent the co-expressions of endothelial markers CD31 and mesenchymal marker FSP1 with close proximity on endothelial cells. Endothelial cells with circular arrangement (white arrows) and their surrounding PLA signal dots in the submucosa layer of the urinary bladder of the control (A; *n* = 9) and KC (B; *n* = 11) groups were further selected and counted. (D) Higher-magnification view over the microvessels of KC from panel (B); white bars: 50 μm. (C) Bar graph of PLA dots per endothelial cell count, used for comparison of the two groups (mean PLA dots per cell: KC, 2.32; control, 0.16); the Mann–Whitney U test was applied for statistical analysis.

The mean endothelial cell count (SD) of the KC patients [25.27 (7.05)] was significantly lower than that [37.11 (10.35)] of the control group (*p* = 0.003) (Fig 4). The mean co-expression PLA signal per cell in the KC bladder tissues was significantly higher than that of the control group (Fig 4C).

## Discussion

In the last decade, it has been found that a single subanaesthetic dose of ketamine can block the NMDA receptor, resulting in alleviating depressive symptoms in treatment-refractory patients [20]. Li et al. demonstrated that intraperitoneal injection of a low dosage of ketamine into rats rapidly activated the mammalian target of the rapamycin (mTOR) pathway, thereby increasing the number of synaptic signaling proteins and increasing the number and function of new spine synapses in the prefrontal cortex [21,22]. The ketamine abusers in this study, however, tended to have severe bladder pain after long-term exposure to higher doses of ketamine, possibly resulting from repeated activation of the mTOR pathway. From a study of ketamine-induced cystitis, Baker et al. reported the unique finding of prominent peripheral nerve fasicle hyperplasia with positive antibody labeling for nerve growth factor receptor S100 and neurofilament protein [23]. The possible association between activation of the mTOR pathway and peripheral nerve fascicle hyperplasia might render further investigation.

The NMDA receptor forms a heterotetramer between two obligatory NR1 subunits and two regionally localized NR2 subunits. Baker et al. also recently revealed no expression of NMDAR transcript in human urothelial cells after treatment with ketamine [24]. They further proposed that NMDAR independent pathway characterized by mitochondria stress is the mechanism of direct toxicity to urothelial cell. Based on the discovery of Gonzalez-Cadavid et al., we investigated where exactly NMDAR1 existed in the urinary bladder. In this study, a stronger expression of NMDAR1 was observed in the microvessels of normal human bladders than in those of KC patients, especially in larger vessels (Fig 3). Analogous to the findings in the prefrontal cortex of the mouse, demonstrated by Tang et al., we propose one possible pathway to be the down-regulation of NMDAR1 of microvessels in the urinary bladder of KC patients [25]. The exact underlying mechanism of down-regulation of NMDAR1 and related inflammation remains to be elucidated. Another possibility might be similar to the direct toxicity of urothelial cells upon activating intrinsic apoptosis [24].

Chu et al. hypothesized that the induced microcirculation change in the urinary bladder—an effect of ketamine abuse—might be the result of endothelial cell injury [5]. Thus, for this present study, we used TEM to investigate the correlation between the ultrastructural changes in microcirculation and the clinical manifestations in KC and control groups. We observed a significant morphological change, a thickening of the BM, in both the greater and small vessels of the KC patients; this change was not evident in the control group. Similar to the manifestation of transplant glomerulopathy under TEM, the BM of vessels taken from the urinary bladders of the KC patients exhibited multi-layer duplication with tortuous and angular changes to the vascular lumen [13]. Xu-Dubois et al. demonstrated that antibody-mediated rejection is the prototypical model of endothelial injury in transplant glomerulopathy and that endothelial mesenchymal transition (EndoMT) markers can be used as a diagnostic tool for detecting endothelial cell activation and subsequent fibrosis [26]. In our present study, the layered appearance of the BM (not observed in the normal control group) suggests that endothelial cell activation and MVI occurred in the microcirculation after activation by ketamine.

To investigate whether the vessels are primary target sites or secondary reactions to inflammatory cells, we theorized that the expression of NMDAR1 on the endothelial cells in the KC and control groups might reveal the possible site that initiates the cascade of inflammatory reaction and fibrotic change. Ketamine-mediated activation of NMDAR1 on the endothelial cells may contribute to the thickening of the BM and chronic inflammation with interstitial fibrosis over the submucosal layer. Another type of cellular transition, EndoMT, has emerged as a crucial method of pathogenesis in tissue fibrosis [27–31]. Fibroblasts and myofibroblasts can be activated from an affected endothelium with dedifferentiation. In our present study, we investigated the dedifferentiation of the endothelial cells as a means of activating fibroblasts or myofibroblasts through the expression of mesenchymal markers (FSP-1, α-SMA) in cells featuring co-localization of the endothelial lineage specific cell surface marker CD-31. By using PLA to precisely compare the expression of EndoMT in the vessels, we found that the presence of EndoMT in the KC patients was statistically stronger than that in the control groups. The involvement of EndoMT in the process of fibrosis of the bladder wall in the KC patients might partly account for their diminished bladder capacity relative to that of the control group [6].

Nonetheless, the manifestation through which inflammatory cells play the most important role in triggering the signaling pathway of fibrotic change has yet to be established. Another hypothesis is a potential relationship between KC and mast cells or eosinophil and immune complexes (immunoglobulin E) accumulated over arterioles [6,8]. Jhang et al. reported that histological findings of bladder biopsies of KC revealed many eosinophils and mast cells in the suburothelial tissue and fibrinoid material in the lumen of arterioles, indicating fibrinoid necrosis in arterioles [8]. The parallel mechanism of asthma airway remodeling, proposed by Asosinph et al., is lung neovascularization followed by an influx of eosinophils, suggesting that angiogenesis is important in the initiation of allergic inflammation; they also demonstrated that nascent endothelial cells in newly forming vessels are sufficient to initiate Th2-inflammation [32]. In our present study, it was also demonstrated that endothelial cell injury is the precursor to inflammatory change and fibrosis. Nevertheless, it will be necessary to perform further investigations into the relationship between eosinophil and endothelial cell injury during the pathogenesis of KC. Because of the illegality of the use of ketamine, many KC patients do not seek treatment until they develop extremely severe conditions, resulting in minimal variation of clinical manifestations. This scenario is the major limitation of our research, and might possibly explain the insignificant correlation between the clinical parameters and the duplication of BM. As a result, it became difficult to establish a statistical correlation between the clinical manifestations of KC and the ultrastructural change of the vessels. Therefore, in subsequent studies it will be necessary to identify the pathophysiological mechanism in animal models, especially with reference to the correlation between endothelial cell injury and eosinophils, and the existence of endothelial dedifferentiation into mesenchymal cells in the urinary bladder.

## Conclusions

This study has demonstrated a significantly greater number of KC patients presenting microvascular remodeling in their urinary bladders than that found within a control group. Accordingly, the pathogenic role of MVI in KC is distinct and unique, and sheds new light on investigations of the underlying mechanism.
